# Molecular Fingerprinting Studies Do Not Support Intrahospital Transmission of *Candida albicans* among Candidemia Patients in Kuwait

**DOI:** 10.3389/fmicb.2017.00247

**Published:** 2017-02-21

**Authors:** Mohammad Asadzadeh, Suhail Ahmad, Noura Al-Sweih, Ziauddin Khan

**Affiliations:** Department of Microbiology, Faculty of Medicine, Kuwait UniversityKuwait, Kuwait

**Keywords:** *Candida albicans*, candidemia, MLST, AFLP, molecular fingerprinting, intrahospital transmission

## Abstract

*Candida albicans*, a constituent of normal microbial flora of human mucosal surfaces, is a major cause of candidemia in immunocompromised individuals and hospitalized patients with other debilitating diseases. Molecular fingerprinting studies have suggested nosocomial transmission of *C. albicans* based on the presence of clusters or endemic genotypes in some hospitals. However, intrahospital strain transmission or a common source of infection has not been firmly established. We performed multilocus sequence typing (MLST) on 102 *C. albicans* bloodstream isolates (representing 92% of all culture-confirmed candidemia patients over a 31-month period at seven major hospitals) to identify patient-to-patient transmission or infection from a common source in Kuwait, a small country in the Middle East where consanguineous marriages are common. Repeat bloodstream isolates from six patients and nine surveillance cultures from other anatomic sites from six patients were also analyzed. Fifty-five isolates belonged to unique genotypes. Forty-seven isolates from 47 patients formed 16 clusters, with each cluster containing 2–9 isolates. Multiple isolates from the same patient from bloodstream or other anatomical sites yielded identical genotypes. We identified four cases of potential patient-to-patient transmission or infection from a common source based on association analysis between patients' clinical/epidemiological data and the corresponding MLST genotypes of eight *C. albicans* isolates. However, further fingerprinting by whole genome-based amplified fragment length polymorphism (AFLP) analysis yielded 8 different genotypes, ruling out intrahospital transmission of infection. The findings suggest that related strains of *C. albicans* exist in the community and fingerprinting by MLST alone may complicate hospital infection control measures during outbreak investigations.

## Introduction

*Candida* and other yeast species, like many bacteria, are part of normal microbial flora of skin and mucosal surfaces of the gastrointestinal and genitourinary tracts in humans and give rise to opportunistic infections when host defenses are compromised (Kumamoto, 2011; McManus and Coleman, 2014). The isolation of *Candida* species is higher from individuals receiving broad-spectrum antibiotics or corticosteroid treatment or those with other underlying conditions that compromise host immunity such as diabetes, extremes of age (neonates and elderly), pregnancy and human immunodeficiency virus (HIV) infection (Vincent et al., 2009; Liu et al., 2015; Sun et al., 2016). Typically, these conditions also predispose the colonized individuals to invasive infections by *Candida* species (Vincent et al., 2009; Kett et al., 2011). Although, many *Candida* species are of clinical importance in humans, *Candida albicans* is the most prevalent and most pathogenic species causing the majority of cases of oral and systemic candidiasis as well as candidemia in hospitalized patients (Leroy et al., 2009; Neofytos et al., 2010; Pfaller et al., 2011; Zomorodian et al., 2011). Although, *C. dubliniensis* is another yeast capable of forming true hyphae, it is significantly less pathogenic, causing invasive infections much less frequently than *C. albicans* (Stokes et al., 2007; Moran et al., 2012).

The epidemiology and origin of nosocomial *C. albicans* among candidemia patients can be studied by molecular characterization and fingerprinting of single isolates from individual patients by highly effective molecular typing systems capable of discriminating closely related but non-identical isolates (McManus and Coleman, 2014). Multilocus microsatellite typing (MLMT) based on variations in stretches of tandemly repeated sequences and multilocus sequence typing (MLST) based on single nucleotide polymorphisms in DNA fragments of housekeeping genes with discriminatory powers of 0.987 and 0.999, respectively, are comparable to the more tedious Ca3-based fingerprinting (discriminatory power of 0.93) and are considered sufficient to discriminate even closely related strains (Bougnoux et al., 2003; Tavanti et al., 2003; Sampaio et al., 2005; Odds and Jacobsen, 2008). Of these, MLST is a standardized scheme based on 7 conserved housekeeping genes, is slightly more discriminatory, and has a publicly accessible and curated online *C. albicans* MLST database for worldwide comparisons (http://pubmlst.org/calbicans/). As of December 23, 2016, 4318 *C. albicans* isolates have been classified into 3268 diploid sequence types (DSTs) belonging to 18 different clades (McManus and Coleman, 2014; http://pubmlst.org/calbicans/). Although, MLMT and MLST have high discriminatory power, they are considered inferior to genome wide techniques particularly whole genome sequencing or whole genome multilocus sequence typing (Fitzpatrick et al., 2016; Kwong et al., 2016; Roisin et al., 2016). Previous studies based on MLMT and MLST have reported the presence of endemic genotypes of *C. albicans* within the same hospital units that were likely involved in intrahospital transmission of infection, however, patient-to-patient transmission of infection was not conclusively proven (Asmundsdóttir et al., 2008; Maganti et al., 2011; Shin et al., 2011; Escribano et al., 2013; Marcos-Zambrano et al., 2015; Wu et al., 2015). Patient-to-patient intrahospital transmission of infection was also presumed for nosocomial bacterial pathogens like *Staphylococcus aureus* based on fingerprinting by MLST (McBryde et al., 2004; SenGupta et al., 2014; Ugolotti et al., 2016). However, whole genome sequence-based high-resolution genetic analyses did not support intrahospital transmission of *S. aureus* between patients with invasive infections. These studies also showed that highly related pools of bacterial strains exist which could not be differentiated by low-resolution typing methods such as MLST (Nübel et al., 2013; Long et al., 2014; Price et al., 2014). It is, therefore, probable that the presumed intrahospital transmission of *C. albicans* described in some studies may have been inferred from low-resolution typing methods such as MLMT or MLST which may not have been supported by high-resolution genetic analyses based on whole genome sequencing studies. Since whole genome sequencing is not yet routine for fungi, an alternative approach may involve initial fingerprinting by MLST followed by other whole genome-based high-resolution fingerprinting methods on epidemiologically-related isolates for detecting possible patient-to-patient transmission of infection or infection from a common source.

This study performed molecular fingerprinting of *C. albicans* isolated from 102 candidemia patients (representing 92% of all culture-confirmed candidemia patients at seven major hospitals) in Kuwait by MLST. Potential cases of nosocomial transmission of infection based on epidemiological data and identical MLST genotypes were further studied by fingerprinting by whole genome-based amplified fragment length polymorphism (AFLP) analyses for confirmation of intrahospital transmission of infection.

## Materials and methods

### Reference strains and clinical isolates

Reference strains of *C. albicans* (ATCC 90028), *Candida parapsilosis* (ATCC 22019), *C. parapsilosis* (CBS1954), and *C. parapsilosis* (CBS6318) were used in this study. A total of 171 candidemia episodes were recorded among 111 patients at the seven major hospitals in Kuwait during January 2011 to July 2013. Of these, first blood culture *C. albicans* isolates from 102 patients were available for fingerprinting studies. Additionally, repeat (duplicate) bloodstream *C. albicans* isolates from six (patient no. 2, 5, 26, 60, 73, and 92) patients and nine surveillance cultures from six (patient no. 26, 33, 48, 53, 73, and 83) patients were also included. Blood and other clinical specimens were collected at various hospitals across Kuwait after obtaining verbal consent from patients as part of routine patient care for the isolation of fungal pathogens. The isolates were sub-cultured on Sabouraud dextrose agar medium for identification and antifungal susceptibility testing and data were analyzed anonymously. The consent procedure and the study were approved by the Joint Committee for the Protection of Human Subjects in Research, Health Sciences Center, Kuwait University and Ministry of Health, Kuwait.

The age of the patients ranged from newly born to 106 years. Ninety patients were Kuwaiti nationals while only 12 patients were expatriates originating from four different countries (Supplementary Table 1). Majority (70, 69%) of the patients were adults while 24 of the 32 pediatric patients were neonates. The highest number of *C. albicans* were analyzed from Mubarak Al-Kabeer Hospital (*n* = 27) followed by Maternity Hospital (*n* = 21), Ibn-Sina Hospital (*n* = 17), Farwaniya Hospital (*n* = 14), Al-Sabah Hospital (*n* = 8), Jahra Hospital (*n* = 8) and Amiri Hospital (*n* = 7). Only three (Maternity Hospital, Ibn-Sina Hospital and Al-Sabah Hospital) of these hospitals are within 1 km of each other while the remaining hospitals are situated >10 km apart. All clinical isolates were initially identified as *C. albicans* by commercial CHROMagar Candida and Vitek2 yeast identification system (bioMérieux, Marcy-lEtoile, France). Genomic DNA from each isolate was prepared as described previously (Ahmad et al., 2004). The identity of each isolate was confirmed by duplex PCR targeting the ITS region of rDNA and/or by DNA sequencing of rDNA, as described previously (Ahmad et al., 2012).

### Antifungal drug susceptibility testing

The susceptibility of *C. albicans* isolates against antifungal drugs, amphotericin B (AMB), fluconazole (FLU), voriconazole (VOR), and caspofungin (CFG) was determined by E-test (AB Biodisk, Solna, Sweden) on RPMI agar (supplemented with 2% glucose and buffered with MOPS, 0.165 M, pH 7.0) plates according to manufacturer's recommendations and as described previously (Asadzadeh et al., 2008). Briefly, each isolate was resuspended in sterile normal saline and the turbidity was adjusted to 0.5 McFarland unit. The plates were inoculated with the uniform suspension by using a cotton swab, Etest strips were applied, the plates were incubated at 35°C and read after 24 h. The minimum inhibitory concentration (MIC) values (μg/ml) were recorded for each drug and MIC50 and MIC90 values were also calculated. The revised interpretive susceptibility breakpoints as recommended by Clinical Laboratory Standards Institute (CLSI) were used for FLU (≤2 μg/ml, susceptible; 4 μg/ml, susceptible dose-dependent and ≥8 μg/ml, resistant), VOR (≤0.125 μg/ml, susceptible; 0.25–0.5 μg/ml, intermediate and ≥1 μg/ml, resistant) and CFG (≤0.25 μg/ml, susceptible; 0.5 μg/ml, intermediate and ≥1 μg/ml, resistant) (Clinical and Laboratory Standards Institute, 2012). Due to lack of defined breakpoints for AMB, an isolate showing MIC ≤1.0 μg/ml was considered as susceptible. Quality control was ensured by testing *C. albicans* ATCC 90028 and *C. parapsilosis* ATCC 22019, as recommended by CLSI.

### Molecular fingerprinting of *C. albicans* isolates by MLST

A total of seven (AAT1, ACC1, ADP1, MPI1, SYA1, VPS13, and ZWF1b) housekeeping gene fragments recommended as an international standard for *C. albicans* MLST were used, as described previously (Bougnoux et al., 2003). The oligonucleotide primers (sequences of primers are publicly available at the *C. albicans* MLST database homepage (http://pubmlst.org/calbicans/) were synthesized and used for PCR amplification of the seven genes. Amplification of each gene fragment was performed by PCR using genomic DNA from each isolate, as described previously (Bougnoux et al., 2003). The amplicons were purified by using PCR product purification kit according to the instructions supplied by the kit manufacturer (Qiagen). Both strands were sequenced by using BigDye terminator DNA sequencing kit (ABI Prism BigDye terminator v3.1, Applied Biosystems) using the same primers as used for PCR amplification for each DNA fragment. The sequencing products were processed and loaded on the capillaries of the DNA sequencer (ABI Prism 3130xl Genetic Analyser) as directed by the manufacturer of the automated DNA sequencer (Applied Biosystems) and as described previously (Asadzadeh et al., 2015). Each allele profile was checked in both forward and reverse sequence by using Clustal omega (http://www.ebi.ac.uk/Tools/msa/clustalo/) and each sequence chromatogram was reviewed for heterozygous (two equally strong and overlayed fluorescence peaks) nucleotide positions. The allele number and diploid sequence type (DST) for each isolate was assigned according to *C. albicans* database. The allelic profiles that could not be assigned to an existing DST were consequently added to the MLST database (http://pubmlst.org/calbicans/).

Based on the allele number for the seven gene fragments for each isolate, a dendrogram was constructed by using BioNumerics v7.5 software (Applied Maths, Sint-Martens-Latem, Belgium) and standard unweighted pair group method with arithmetic mean (UPGMA) settings. The genetic relationship between the genotypes was studied by constructing a minimum spanning tree. The isolates were considered belonging to the same DST when they contained the same alleles for all seven loci. A cluster was defined as the same DST causing bloodstream infection in two or more candidemia patients.

### AFLP analysis of similar and epidemiologically related *C. albicans* isolates

Cluster *C. albicans* isolates with identical MLST-based genotype (DST) and epidemiological linkage with respect to their isolation from the same hospital within a period of 60 days were further analyzed by AFLP analysis. A total of eight *C. albicans* isolates in four clusters (2 isolates in each), all isolated from candidemia patients in 2011, were used. The AFLP analysis was carried out as described in detail previously (Illnait-Zaragozí et al., 2012). Briefly, 50 ng of genomic DNA was mixed with restriction-ligation mixture containing restriction enzymes EcoR1 and MseI (New England Biolabs, Beverly, MA, USA) and their corresponding complementary adaptors. Prior to further use, the restriction-ligation reaction was diluted by adding 80 μl of 10 mM Tris-HCl (pH 8.3) buffer and one microliter of the diluted product was used for amplification in a total volume of 25 μl by using the selective primers EcoR1 (5′-FLU-GACTGCGTACCAATTCAC-3′) and MseI (5′-GATGAGTCCTGACTAAC-3′) (Illnait-Zaragozí et al., 2012). One microliter of a 10-fold dilution of amplified products was added to a mixture of 8.9 μl water and 0.1 μl LIZ600 internal size marker (Applied Biosystems) and the contents were loaded for fragment analysis on an ABI 3500xL Genetic Analyzer according to the instructions of the manufacturer (Applied Biosystems). *C. albicans* reference strain (ATCC 90028) was used as a control and three reference strains (ATCC22019, CBS1954, and CBS6318) of *C. parapsilosis* were used as an out group. Raw data were analyzed by using BioNumerics v6.6 software and a dendrogram was generated using standard Pearson and unweighted pair group method with arithmetic mean (UPGMA) settings. A cut-off value of ≤95% similarity index among AFLP patterns was used for defining a distinct genotype (Asadzadeh et al., 2015).

### Statistical analysis

Categorical variables were expressed as absolute number. Statistical analysis was performed using chi-square test or Fisher's exact test as appropriate and probability levels <0.05 by the two-tailed test were considered as statistically significant. Statistical analyses were performed using WinPepi software ver. 3.8 (PEPI for Windows, Microsoft Inc., Redmond, WA, USA).

## Results

### Distribution of episodes of candidemia at seven major hospitals in Kuwait

Kuwait is a small Arabian Gulf country with nearly 1.3 million Kuwaiti and a larger (~3 million) ethnically diverse but mostly younger (20–50 year) and medically screened expatriate population. All residents receive nearly free medical treatment in government-run hospitals. Nearly 40–50 culture-confirmed cases of candidemia due to *C. albicans* occur every year at seven of the nine major/tertiary-care hospitals of the Ministry of Health, Government of Kuwait, where candidemia patients are mainly diagnosed and treated. These seven hospitals serve a large (~85%) proportion of Kuwait's multinational population and the location of these hospitals within Kuwait is shown in Supplementary Figure 1. During the 31-month (January 2011 to July 2013) study period, 111 culture-confirmed candidemia patients due to *C. albicans* were treated at these seven hospitals (Table 1). The highest number of candidemia cases were diagnosed and treated at Mubarak Al-Kabeer Hospital (*n* = 29) followed by Maternity Hospital (*n* = 22), Ibn-Sina Hospital (*n* = 19) and Farwaniya Hospital (*n* = 14) while the remaining 27 patients were treated at the remaining three hospitals. A total of 102 *C. albicans* bloodstream isolates recovered from 102 candidemia patients (representing 92% of all individual patient isolates from seven hospitals) were available for fingerprinting studies (Table 1). Most (90, 88%) of the patients were Kuwaiti nationals while only 12 patients were expatriates originating from four different countries (Supplementary Table 1). Majority (70, 69%) of the patients were adults while 24 of the 32 pediatric patients were neonates. All patients from Maternity Hospital (*n* = 21) were admitted to an intensive care unit (ICU) and mostly (17 of 21) included pre-term low birth-weight neonates on total parenteral nutrition. Overall, 47 of 102 bloodstream isolates were obtained from patients in an intensive care unit (Supplementary Table 1). For some patients, duplicate or multiple isolates were grown from repeated blood samples, typically within 1 week of isolation of the first blood culture. Surveillance cultures from different anatomic sites were also obtained from hospitalized patients. Thirty-two (31%) patients had multiple bloodstream isolates, including 12 patients with two isolates, 16 patients with three isolates, three patients with four isolates and one patient with five isolates. Duplicate bloodstream isolates from six patients and 9 surveillance cultures from other anatomic sites from six patients were also included for MLST analyses to determine whether *C. albicans* isolates causing candidemia or multiple episodes of candidemia were genetically similar or distinct strains and their relationship with colonizing strains. All 117 isolates were identified as *C. albicans* by the Vitek 2 yeast identification system, CHROMagar Candida and a duplex PCR assay or by PCR sequencing of rDNA (Ahmad et al., 2012).

**Table 1:** **Number of culture confirmed candidemia episodes recorded at seven major hospitals in Kuwait and number of *C. albicans* isolates analyzed by MLST**.

**Hospital**	No. of candidemia episodes^*^	**No. of patients**	**No. of individual patient isolates available for MLST**	**No. of duplicate and surveillance cultures used for MLST**
Mubarak Al-Kabeer	43	29	27	1
Maternity	50	22	21	9
Ibn-Sina	25	19	17	2
Farwaniya	16	14	14	−
Al-Sabah	15	9	8	2
Jahra	10	9	8	−
Amiri	12	9	7	1
Total	171	111	102	15

* *Repeat isolates were cultured from some candidemia patients*.

### Antifungal susceptibility testing

All 102 bloodstream *C. albicans* isolates were tested for *in vitro* antifungal drug susceptibility against four antifungal drugs, viz. fluconazole (FLU), voriconazole (VOR), amphotericin B (AMB) and caspofungin (CSF) by Etest. The distribution of minimum inhibitory concentration (MIC) values of 102 *C. albicans* isolates are summarized in Table 2. A total of 101 isolates were susceptible to all four antifungal agents according to the CLSI breakpoints for *C. albicans* while one isolate (Kw1104) was resistant to both fluconazole and voriconazole but susceptible to amphotericin B and caspofungin. The duplicate isolates as well as the nine surveillance cultures were susceptible to all antifungal drugs tested.

**Table 2:** ***In vitro*** **susceptibility of 102 bloodstream *C. albicans* isolates against four antifungal drugs**.

**Antifungal agent**	**Minimum inhibitory concentration (MIC, μg/ml)**		
	**Range**	**MIC_50_**	**MIC_90_**
Amphotericin B	0.002–0.75	0.047	0.125
Fluconazole	0.023–>256	0.38	0.75
Voriconazole	0.002–>32	0.016	0.25
Caspofungin	0.002–0.38	0.125	0.25

*MIC_50_ and MIC_90_, MIC required to inhibit 50% and 90% of the isolates, respectively*.

*One isolate (Kw1104) belonging to DST1056 was resistant to both fluconazole and voriconazole*.

### Fingerprinting of *C. albicans* isolates by MLST

Fingerprinting of bloodstream *C. albicans* isolates was carried out to ascertain patient-to-patient or nosocomial transmission of infection in hospitalized candidemia patients. For this purpose, *C. albicans* isolates yielding unique DST were considered as genotypically distinct strains while isolates yielding the same DST were considered as candidates for possible patient-to-patient or nosocomial transmission of infection. However, patient-to-patient or nosocomial transmission of infection was considered as likely only if the two isolates yielding identical DST were related with respect to both, time and space, i.e., they were collected from two or more patients from the same hospital and within a short period of time. An arbitrary window period of ≤60 days was used to define epidemiologically related isolates with identical DST collected from two or more patients from the same hospital. The location of the hospitalized patients in different units was not used to exclude epidemiological linkage since patients are often moved within the hospital and this information is not always readily available.

Fingerprinting of 102 *C. albicans* bloodstream isolates from 102 patients identified 71 different DSTs including six novel alleles (three new AAT1α alleles, two MPIb alleles and one VPS13 allele) identified for the first time in this study. Thirty-seven of 71 DSTs have been identified previously and described in the MLST database (http://pubmlst.org/calbicans/) while 34 DSTs have been identified for the first time (Supplementary Table 1). Thirty-one of the 34 new DSTs were represented by bloodstream isolates from 31 different patients while three new DSTs (DST2286, DST2289, and DST2292) were obtained from six different patients in three clusters and in each case, the two patients infected with the same genotype were treated in two different hospitals. Among the four major hospitals, the ratio of new DSTs detected among total DSTs was nearly same (~50%) in Mubarak Al-Kabeer hospital (10 of 23, 43%), Ibn-Sina Hospital (7 of 13, 54%) and Farwaniya Hospital (7 of 14, 50%) but was lower from Maternity Hospital (4 of 17, 24%), however, the difference was not statistically significant. Similarly, the ratio of new DSTs detected among total DSTs in expatriate patients (5 of 12, 42%), and Kuwaiti patients (29 of 66, 44%) was also nearly same (Supplementary Table 1). The *C. albicans* isolate (Kw1104) resistant to fluconazole and voriconazole belonged to DST1056. The newly identified alleles and DSTs have been submitted to the publicly available *C. albicans* MLST database (http://pubmlst.org/calbicans/).

**Figure 1:**
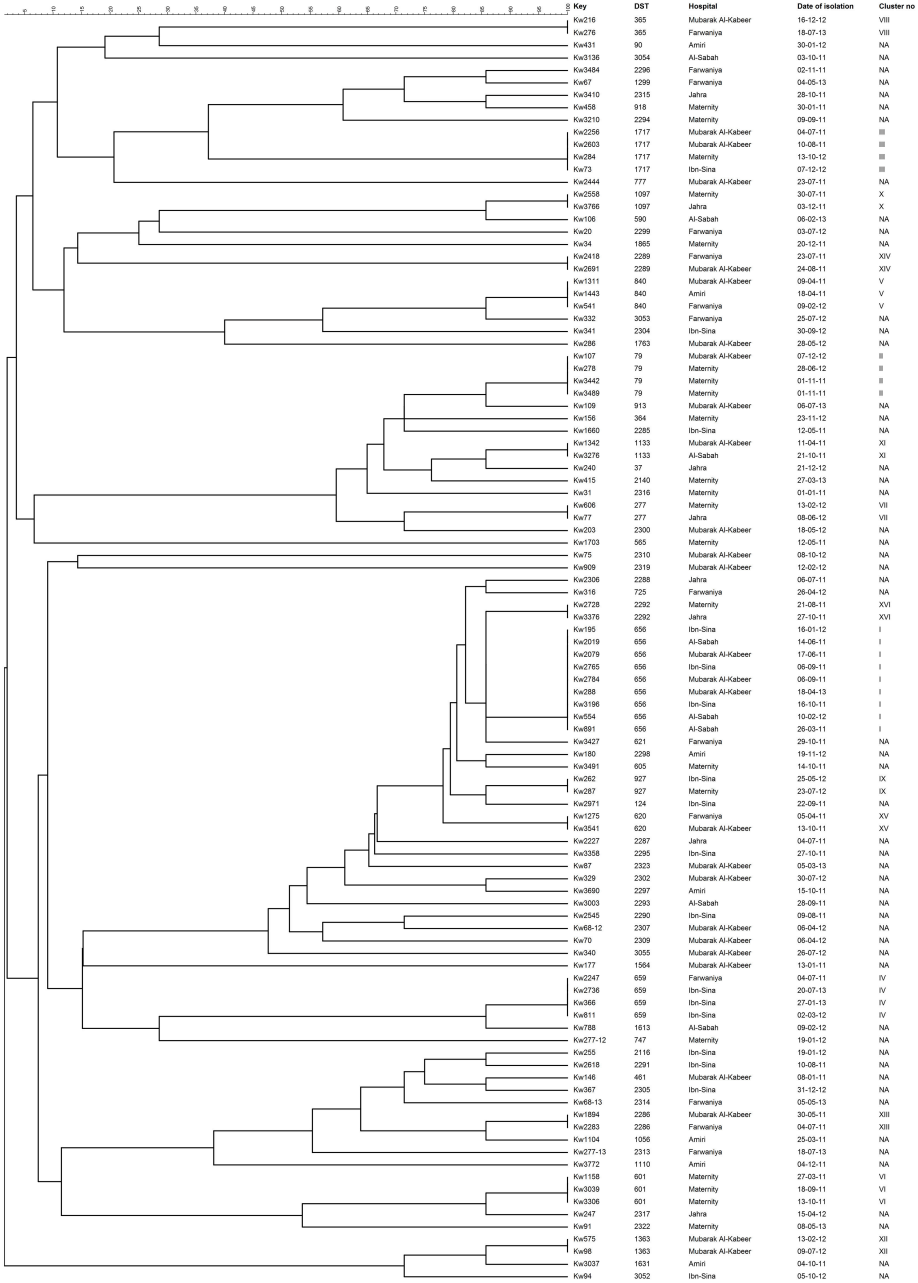
**An UPGMA-derived dendrogram based on allele numbers of seven housekeeping gene fragments from 102 bloodstream *C. albicans* isolates from 102 patients**. Similarity is presented in percentages using the scale bar in the upper left corner. The columns after the isolate numbers refer to the MLST-based DSTs, name of hospital where candidemia patients were hospitalized, date of culture of the isolate and cluster number if two or more isolates belonged to the same DST. NA, not applicable.

**Figure 2:**
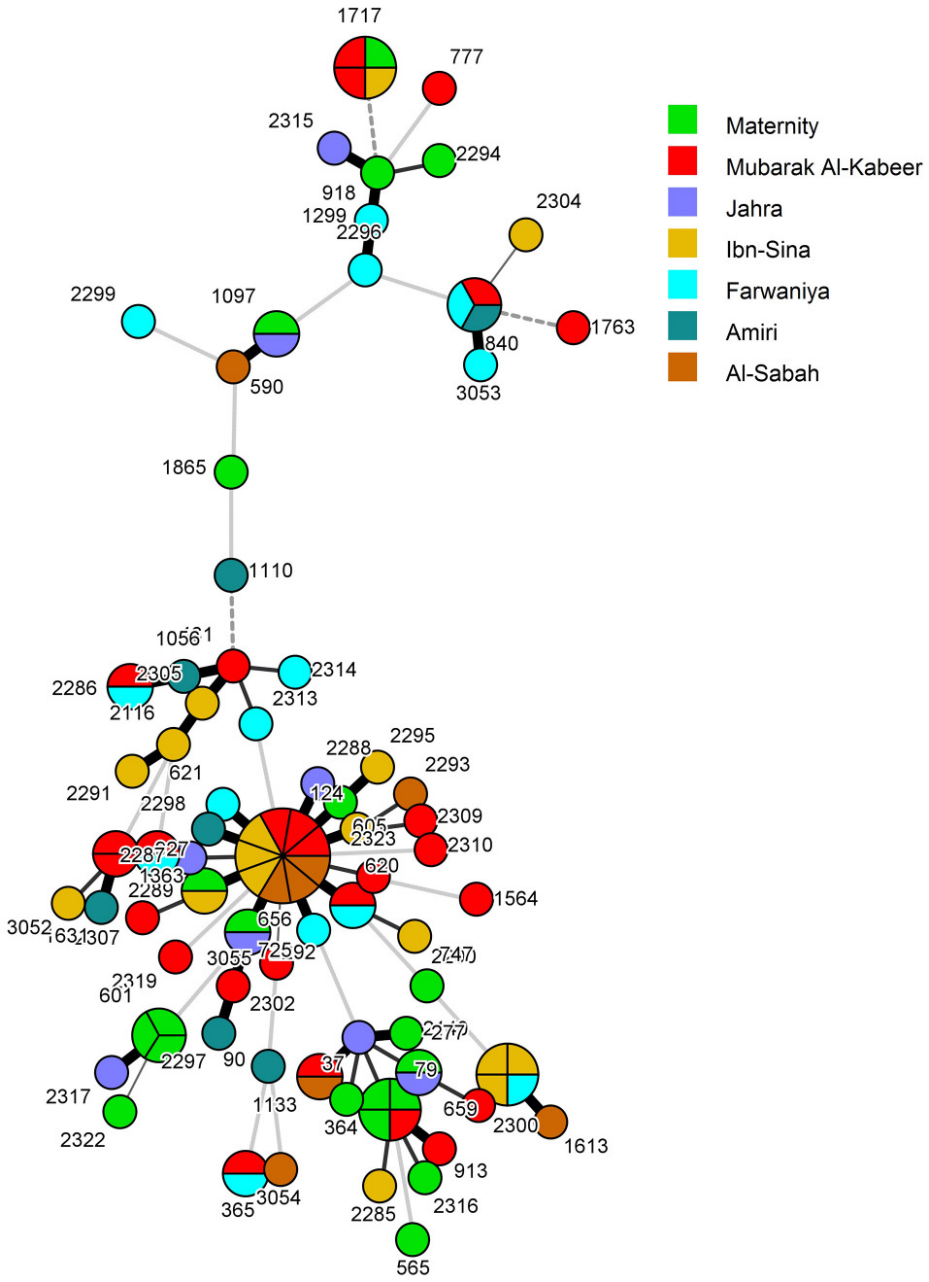
**Minimum spanning tree of 102 *C. albicans* isolates derived from MLST data**. Each circle corresponds to a unique genotype, and lines between circles represent relative distance between isolates. The sizes of the circles correspond to the number of isolates of the same genotype (DST). The numbers refer to the MLST-based DSTs. Connecting lines correspond to the number of differences between genotypes, with a solid thick line connecting genotypes that differ in one locus, a solid thin line connecting genotypes that differ in two-three loci, a dashed line connecting genotypes that differ in four loci, and a dotted line connecting genotypes that differ in more than four loci.

Overall, 55 *C. albicans* isolates from 55 patients belonged to 55 unique DSTs (including 31 new DSTs) while 47 isolates from 47 patients were clustered in 16 DSTs. The largest cluster represented by DST656 contained nine isolates from nine patients hospitalized in three hospitals followed by three other clusters (represented by DST79, DST659, and DST1717) with each cluster shared by four isolates and involving patients from 2 to 3 hospitals (Figure 1 and Table 3). Two other clusters (represented by DST601 and DST840) contained three isolates each, with DST601 yielding *C. albicans* isolates from Maternity Hospital only. The remaining 10 clusters contained only two isolates in each cluster and the two patients yielding *C. albicans* isolates in only one cluster (DST1133) were hospitalized in the same hospital (Figure 1 and Table 3). The analysis of MLST data also showed that patients in nearly all clusters were distributed across various hospitals in Kuwait (Figure 2). Repeat bloodstream isolates as well as surveillance cultures obtained from different anatomical sites yielded the same MLST genotype as the first bloodstream isolate recovered from the same patient (data not shown).

**Table 3:** **Date and place of isolation of *C. albicans* isolates yielding identical multilocus sequence type (MLST)-based diploid sequence types (DSTs) in 16 clusters**.

**Patient no**.	**Isolate no**.	**Date of isolation (DD-MM-YY)**	**Hospital**	**Hospital ward^a^**	**MLST-based DST**	**Cluster no.^e^**
6	Kw891	26-03-11	Al-Sabah	ICU	656^b^	I
15	Kw2019	14-06-11	Al-Sabah	PICU	656^b^	I
60	Kw554	10-02-12	Al-Sabah	1	656^b^	I
16	Kw2079	17-06-11	Mubarak Al-Kabeer	11	656^b^	I
31	Kw2784	06-09-11	Mubarak Al-Kabeer	9	656^b^	I
95	Kw288	18-04-13	Mubarak Al-Kabeer	17	656^b^	I
**30**	**Kw2765**	**06-09-11**	**Ibn-Sina**	**ICU**	**656**^b^	**I**
**42**	**Kw3196**	**16-10-11**	**Ibn-Sina**	**7**	**656**^b^	**I**
54	Kw195	16-01-12	Ibn-Sina	PICU	656^b^	I
**48**	**Kw3442**	**01-11-11**	**Maternity**	**NICU-2**	**79**^c^	**II**
**49**	**Kw3489**	**01-11-11**	**Maternity**	**NICU-1**	**79**^c^	**II**
73	Kw278	28-06-12	Maternity	NICU-1	**79**^c^	II
86	Kw107	07-12-12	Mubarak Al-Kabeer	ICU	**79**^c^	II
**18**	**Kw2256**	**04-07-11**	**Mubarak Al-Kabeer**	**24**	**1717**^b^	**III**
**27**	**Kw2603**	**10-08-11**	**Mubarak Al-Kabeer**	**23**	**1717**^b^	**III**
83	Kw284	13-10-12	Maternity	NICU-1	1717^b^	**III**
87	Kw73	07-12-12	Ibn-Sina	7	1717^b^	**III**
17	Kw2247	04-07-11	Farwaniya	CCU	659^b^	IV
64	Kw811	02-03-12	Ibn-Sina	Medical	659^b^	IV
91	Kw366	27-01-13	Ibn-Sina	7	659^b^	IV
101	Kw2736	20-07-13	Ibn-Sina	ICU	659^b^	IV
9	Kw1311	09-04-11	Mubarak Al-Kabeer	12	840^b^	V
11	Kw1443	18-04-11	Al-Amiri	ICU	840^b^	V
58	Kw541	09-02-12	Farwaniya	NICU-8	840^b^	V
7	Kw1158	27-03-11	Maternity	SCU-2	601^b^	VI
**33**	**Kw3039**	**18-09-11**	**Maternity**	**NICU-1**	**601**^b^	**VI**
**39**	**Kw3306**	**13-10-11**	**Maternity**	**SCU-1**	**601**^b^	**VI**
62	Kw606	13-02-12	Maternity	SCU-1	277^c^	VII
72	Kw77	08-06-12	Jahra	SCU-1	277^c^	VII
88	Kw216	16-12-12	Mubarak Al-Kabeer	12	365^b^	VIII
100	Kw276	18-07-13	Farwaniya	8	365^b^	VIII
8	Kw1275	05-04-11	Farwaniya	NICU-8	620^b^	IX
38	Kw3541	13-10-11	Mubarak Al-Kabeer	22	620^b^	IX
70	Kw262	25-05-12	Ibn-Sina	7	927^b^	X
76	Kw287	23-07-12	Maternity	NICU-1	927^b^	X
24	Kw2558	30-07-11	Maternity	NICU-1	1097^b^	XI
51	Kw3766	03-12-11	Jahra	6	1097^b^	XI
10	Kw1342	11-04-11	Mubarak Al-Kabeer	ICU	1133^b^	XII
43	Kw3276	21-10-11	Al-Sabah	1	1133^b^	XII
63	Kw575	13-02-12	Mubarak Al-Kabeer	Medical	1363^b^	XIII
75	Kw98	09-07-12	Mubarak Al-Kabeer	ICU	1363^b^	XIII
14	Kw1894	30-05-11	Mubarak Al-Kabeer	22	2286^d^	XIV
19	Kw2283	04-07-11	Farwaniya	NICU-8	2286^d^	XIV
23	Kw2418	23-07-11	Farwaniya	SCU-B	2289^d^	XV
29	Kw2691	24-08-11	Mubarak Al-Kabeer	26	2289^d^	XV
28	Kw2728	21-08-11	Maternity	NICU-1	2292^d^	XVI
44	Kw3376	27-10-11	Jahra	27	2292^d^	XVI

a *ICU, intensive care unit; PICU, pediatric intensive care unit; NICU, neonatal intensive care unit; SCU, special care unit; CCU, coronary care unit*.

b *Rare genotypes*.

c *Relatively more common genotypes*.

d *New genotypes found in this study*.

e *Cluster isolates with identical DST and epidemiological linkage are shown in bold*.

When the patient's clinical and epidemiological data, date of isolation of the *C. albicans* isolate and the hospital where the patients were treated were considered, only four clusters involving two isolates in each cluster were identified which could potentially represent patient-to-patient or nosocomial transmission of infection. In each case, both patients (including 4 neonates) were infected with relatively rare DSTs, developed candidemia within a span of 46 days and were admitted to the same intensive care unit/ward and/or were attended by the same heath care personnel suggesting cross-transmission of infection (Table 3). The remaining cluster isolates were epidemiologically unrelated as they were recovered from patients hospitalized in different hospitals and/or were recovered at considerably different time points even from patients treated within the same hospital.

### Fingerprinting of cluster *C. albicans* isolates by AFLP analysis

**Figure 3:**
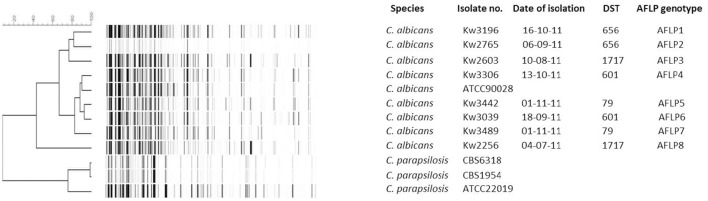
**An UPGMA-derived dendrogram based on AFLP fingerprints for eight *C. albicans* isolates from eight patients found in four DST clusters**. The reference *C. albicans* strain (ATCC 90028) was also included in AFLP analysis and three reference strains of *C. parapsilosis* (CBS 6318, CBS 1954, ATCC 22019) were used as outer groups. Similarity is presented in percentages using the scale bar in the upper left corner. The columns after the species represent isolate number, date of isolation, DST, and arbitrarily defined AFLP genotype.

A total of eight (Kw3196, Kw2765, Kw2603, Kw3306, Kw3442, Kw3039, Kw3489, and Kw2256) *C. albicans* isolates from eight patients in four DST (DST79, DST601, DST656, and DST1717) clusters were subjected to further fingerprinting by amplified fragment length polymorphism (AFLP). Reference *C. albicans* (ATCC90028) and *C. parapsilosis* (CBS6318, CBS1954, and ATCC22019) strains were also used for comparisons. Three reference strains of *C. parapsilosis* were used to confirm the robustness of AFLP data. Two *C. parapsilosis* strains (CBS6318 and CBS1954) are extremely closely related strains sharing 98.9% identity while *C. parapsilosis* ATCC22019 is distantly related to CBS6318 and CBS1954 and shares only 76% identity. The results of AFLP confirmed these data as the AFLP patterns for *C. parapsilosis* CBS6318 and CBS1954 were >98% identical. The eight *C. albicans* isolates yielded a total of 115 AFLP fragments of which 95 were polymorphic. An arbitrary cut-off value of ≤95% similarity among AFLP patterns was used for defining a distinct genotype. All eight isolates yielded eight different AFLP fingerprinting patterns and eight distinct AFLP genotypes (labeled as AFLP1 to AFLP8) (Figure 3). The cluster isolates exhibited 44 to 87% identity among each other based on AFLP patterns. Interestingly, isolate Kw2603 belonging to DST1717 exhibited closer relationship with the isolates (Kw3196 and Kw2765) in DST656 cluster than with isolate Kw2256 belonging to the same (DST1717) genotype. Similar results were obtained for the two (Kw3306 and Kw3039) isolates in DST601 cluster (Figure 3). The AFLP data ruled-out patient-to-patient or nosocomial transmission of infection among candidemia patients in Kuwait.

## Discussion

The genetic diversity among clinical *C. albicans* isolates is investigated by molecular typing techniques amenable to high-throughput capability for epidemiological purposes. These studies have occasionally identified small outbreaks inside hospitals which escaped identification by routine hospital infection control measures (Song et al., 2014; Tsai et al., 2015). This study was carried out for two specific objectives: to generate long-term genetic database of *C. albicans* bloodstream isolates at seven major/tertiary care hospitals and to identify endemic genotypes/cluster isolates that could point toward patient-to-patient or nosocomial transmission of infection among candidemia patients in Kuwait.

Two PCR-based methods, viz. MLST and MLMT have been mostly used in recent years to study global epidemiology, to track outbreaks and to ascertain nosocomial transmission of *C. albicans* infection (Bougnoux et al., 2003; Tavanti et al., 2003, 2005). Most of the molecular epidemiological investigations focusing on the population structure of clinical *C. albicans* isolates have typically relied on fingerprinting patterns by either MLST or MLMT. Based on these analyses, some studies have reported the occurrence of endemic genotypes in some hospitals in few countries treating candidemia patients and have suggested patient-to-patient or nosocomial transmission of infection based on identification of cluster isolates with identical *C. albicans* genotypes (Escribano et al., 2013; Marcos-Zambrano et al., 2015; Tsai et al., 2015; Wu et al., 2015). On the other hand, one recent study failed to observe any epidemiological relationship among cluster isolates with identical DSTs with respect to time of isolation or clinical units within the hospital, thus ruling out nosocomial transmission of infection (Huyke et al., 2015). These contrasting observations could be attributed to the inherent disadvantages associated with the two typing methods. Although, the loci are scattered on different chromosomes, both MLST and MLMT analyze very small regions of *C. albicans* genome in contrast to other techniques such as pulse field gel electrophoresis (PFGE), random amplification of polymorphic DNA (RAPD) and AFLP which potentially examine the entire genome (Ahmad et al., 2003; Borst et al., 2003; Garcia-Hermoso et al., 2007; Odds et al., 2007; McManus and Coleman, 2014). Thus, many closely related but genotypically different isolates may yield identical genotype by MLST or MLMT due to lower discriminatory power of these typing techniques. Consistent with these observations, few studies which analyzed the population structure of *C. albicans* isolates in various settings by more than one molecular fingerprinting technique also seem to support the endogenous origin of most infections (Shin et al., 2011; Ge et al., 2012; Song et al., 2014).

**Figure 4:**
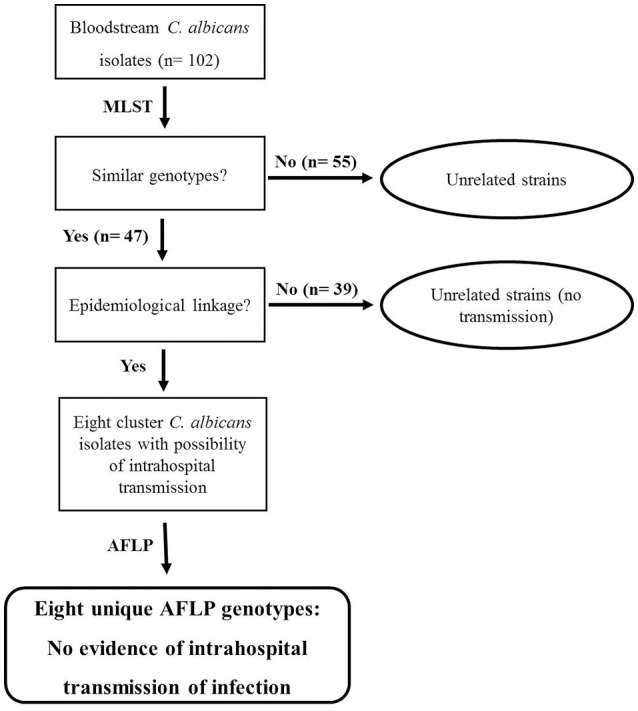
**Flow diagram showing the steps adopted to confirm or rule out intrahospital transmission of *C. albicans* infection among candidemia patients in Kuwait**. Cluster isolates with identical DST cultured from two or more candidemia patients in the same hospital within 60 days were considered possible candidates for intrahospital transmission of infection and were further analyzed by AFLP fingerprinting. No intrahospital transmission of infection was deduced based on eight unique AFLP patterns exhibited by eight MLST-based cluster isolates.

Our initial molecular fingerprinting performed on 102 clinical *C. albicans* bloodstream isolates obtained from 102 candidemia patients admitted in seven major hospitals in Kuwait (representing >92% of all candidemia cases at these hospitals within the 31-month study period) by MLST identified 55 isolates as unique strains (DSTs or genotypes) while 47 isolates were grouped in 16 DSTs (clusters). Repeat bloodstream isolates and surveillance cultures yielded the same MLST genotype as the first bloodstream isolate from each patient. Similar results have also been reported by investigators who have analyzed multiple samples from candidemia patients (Cliff et al., 2008; Afsarian et al., 2015; Moorhouse et al., 2016). Interestingly 12 DSTs were found among 12 *C. albicans* isolates from expatriate candidemia patients originating from four different countries while 64 DSTs (five DSTs were common among the two groups) were found among 90 isolates from Kuwaiti patients. The relatively large number of MLST-based genotypes detected among Kuwaiti candidemia patients was rather surprising since consanguineous marriages common among Kuwaiti nationals are expected to favor selection of relatively fewer *C. albicans* genotypes (Hoben et al., 2010). DST656 was the most common genotype shared among nine isolates. According to the MLST website (http://pubmlst.org/calbicans/), DST656 is a rare genotype comprising isolates originating from only six countries and mostly including isolates from Kuwait and the U.K. Of DST79 and DST1717, each comprising four cluster isolates, DST79 has a worldwide distribution while DST1717 is a minor genotype mainly including isolates from Kuwait and four isolates from three other countries. DST601, DST659, and DST840, each comprising three cluster isolates are also minor genotypes mainly including isolates from Kuwait and a few other countries. These findings suggested that the clustering of some *C. albicans* isolates in rare MLST genotypes more common in some geographical locations may be due to lower discriminatory power of MLST rather than any specific genotypic (clonal) relationship. We therefore, devised a strategy for determining whether cluster isolates represent patient-to-patient or nosocomial transmission of infection among candidemia patients in Kuwait and it is outlined in Figure 4.

The patient's clinical and epidemiological data were reviewed and combined with the date of isolation of the cluster *C. albicans* isolates and the MLST genotype. The *C. albicans* isolates were considered as epidemiologically unrelated strains if the cluster isolates were obtained from candidemia patients in Kuwait from different hospitals or were cultured >60 days apart even if they belonged to a rare MLST-based DST. One study from South Korea has also shown that MLST-based cluster isolates recovered from pairs of patients more than 2 months apart were genotypically distinct strains (Shin et al., 2011). Other investigators have used different cut-off time periods for defining cluster isolates with epidemiological linkage causing bloodstream infections. The window period to define epidemiological relationship with regard to time of isolation of cluster isolates has ranged from ~15 days (Huyke et al., 2015) to 90 days (~3 months) (Tsai et al., 2015). Some investigators have used a 6-month period (Song et al., 2014) or even a much broader window-period that ranged from few days to >1 year (Escribano et al., 2013; Marcos-Zambrano et al., 2015). Based on the criteria defined above for cluster isolates in Kuwait, eight isolates in four clusters were presumed to be epidemiologically linked and represented *C. albicans* isolates with the possibility of intrahospital transmission of infection among candidemia patients (Figure 4). Only two isolates from the largest cluster fulfilled these criteria as the remaining isolates in this cluster were either isolated in different hospitals and/or the duration between their isolation was much longer. However, among the three other clusters, two clusters involved four neonates admitted to the intensive care units of Maternity Hospital.

Additional fingerprinting of cluster isolates by whole genome-based AFLP showed that all eight isolates were distinct strains as cluster isolates exhibited ≤87% identity among each other while clonal strains of *Candida* spp. exhibit >95% identity in AFLP patterns (Chowdhary et al., 2014; Asadzadeh et al., 2015; Schelenz et al., 2016). Furthermore, >82% of AFLP fragments were polymorphic suggesting the occurrence of frequent recombination events among Kuwaiti strains that significantly contributed to the genetic diversity of these isolates during AFLP analyses. Our data also showed that despite the possibility of frequent recombination among the strains, the MLST analyses were not able to detect intra-strain variability, possibly reflecting the limitation of using a very restricted set (only seven housekeeping gene fragments) of gene loci in the currently used MLST scheme for *C. albicans*.

The AFLP analyses ruled out patient-to-patient or nosocomial transmission of infection in Kuwait. The fingerprinting of *C. albicans* isolates from Jena in Germany by MLST identified only two cluster isolates obtained from two neonates from an identical ward and within a short period of 9 days. Epidemiological investigations revealed that the patients were newborn twins strongly suggesting transmission of *C. albicans* from the mother rather than intrahospital transmission of infection (Huyke et al., 2015). Only few studies have performed fingerprinting of *C. albicans* isolates by more than one high-resolution typing techniques. Twenty *C. albicans* isolates obtained from patients with genital candidiasis in China and belonging to DST79 were classified into 11 microsatellite-based (CAI) genotypes showing genotypic heterogeneity among MLST-based cluster. Similarly, 26 cluster isolates belonging to a newly described genotype (DST1867) comprised seven CAI genotypes showing that cluster *C. albicans* isolates belonging to same MLST-based DST are not always clonal (Ge et al., 2012). In two candidemia studies originating from South Korea, initial fingerprinting was performed by MLST followed by PFGE. Interestingly, 48 of 61 (79%) cluster isolates by MLST in one study were reported as unrelated strains by PFGE even though only two cluster isolates shared identical PFGE pattern (Shin et al., 2011). Surprisingly, the remaining 11 isolates were considered as “similar” (clonally related) strains even though they exhibited up to 5% variations in PFGE banding patterns, a finding that should have also placed them as genotypically distinct strains since they belonged to the same DST and are expected, if not clonal, to be closely-related but not identical (Shin et al., 2011). In another study spanning 8 years and involving 43 neonates, only 9 of 28 (32%) cluster isolates by MLST were reported as unrelated strains by PFGE. The *C. albicans* isolates from 14 patients yielded same MLST/PFGE pattern while isolates from five patients exhibited same MLST and similar (defined as ≥95% identical) PFGE pattern (Song et al., 2014). Although, in one case, the two MLST/PFGE cluster (DST182/PFGEtype10a) isolates were cultured 3-years apart, three isolates in one MLST/PFGE cluster (DST79/PFGEtype1), three isolates in a second MLST/PFGE cluster (DST1097/PFGEtype14), three isolates in a third MLST/PFGE cluster (DST2088/PFGEtype29) and two isolates in one MLST/PFGE cluster (DST69/PFGEtype24) were cultured within a short period of time (<50 days). Based on these observations, the authors concluded that horizontal transmission of *C. albicans* occurs more frequently than vertical transmission among neonates hospitalized in NICUs (Song et al., 2014). However, it is also probable that these *C. albicans* isolates were closely related and PFGE could not resolve their genotypic differences. Our results involving 21 neonatal candidemia patients treated in intensive care units of Maternity Hospital in Kuwait support the latter possibility as we did not identify any episode of intrahospital transmission of *C. albicans* infection. The study emphasizes the need of using more sensitive and discriminatory molecular techniques to confirm or rule out intrahospital transmission of *Candida* infection and to develop preventive strategies in the event of presence of endemic genotypes in a given clinical setting. Interestingly, analogous observations have already been made for *S. aureus*, a nosocomial opportunistic bacterial pathogen (Long et al., 2014; Price et al., 2014; Roisin et al., 2016).

A limitation of our study is that the AFLP analyses were not carried out on repeat *C. albicans* isolates from six patients and the nine surveillance cultures that were investigated by MLST.

## Author contributions

Conceived and designed the experiments: MA, SA, NA, and ZK. Performed the experiments: MA. Analyzed the data: MA, SA, NA, and ZK. Contributed reagents/materials/analysis tools: ZK. Wrote the paper: MA, SA, NA, and ZK.

### Conflict of interest statement

The authors declare that the research was conducted in the absence of any commercial or financial relationships that could be construed as a potential conflict of interest.

## References

[B1] Afsarian M. H. Badali H. Boekhout T. Shokohi T. Katiraee F. (2015). Multilocus sequence typing of *Candida albicans* isolates from a burn intensive care unit in Iran. J. Med. Microbiol. 64, 248–253. 10.1099/jmm.0.000015 25596113

[B2] Ahmad S. Khan Z. Asadzadeh M. Theyyathel A. Chandy R. (2012). Performance comparison of phenotypic and molecular methods for detection and differentiation of *Candida albicans* and *Candida dubliniensis*. BMC Infect. Dis. 12:230. 10.1186/1471-2334-12-230 23009343 PMC3541108

[B3] Ahmad S. Khan Z. Mustafa A. S. Khan Z. U. (2003). Epidemiology of Candida colonization in an intensive care unit of a teaching hospital in Kuwait. Med. Mycol. 41, 487–493. 10.1080/1369378031000147458 14725322

[B4] Ahmad S. Mustafa A. S. Khan Z. Al-Rifaiy A. I. Khan Z. U. (2004). PCR-enzyme immunoassay of rDNA in the diagnosis of candidemia and comparison with amplicon detection by agarose gel electrophoresis. Int. J. Med. Microbiol. 294, 45–51. 10.1016/j.ijmm.2004.01.002 15293453

[B5] Asadzadeh M. Ahmad S. Hagen F. Meis J. F. Al-Sweih N. Khan Z. U. (2015). Simple, low-cost detection of *Candida parapsilosis* complex isolates and molecular fingerprinting of *Candida orthopsilosis* strains in Kuwait by ITS region sequencing and amplified fragment length polymorphism analysis. PLoS ONE 10:e0142880. 10.1371/journal.pone.0142880 26580965 PMC4651534

[B6] Asadzadeh M. Al-Sweih N. A. Ahmad S. Khan Z. U. (2008). Antifungal susceptibility of clinical *Candida parapsilosis* isolates in Kuwait. Mycoses 51, 318–323. 10.1111/j.1439-0507.2008.01492.x 18855844

[B7] Asmundsdóttir L. R. Erlendsdóttir H. Haraldsson G. Guo H. Xu J. Gottfredsson M. (2008). Molecular epidemiology of candidemia: evidence of clusters of smoldering nosocomial infections. Clin. Infect. Dis. 47, e17–e24. 10.1086/589298 18549311

[B8] Borst A. Theelen B. Reinders E. Boekhout T. Fluit A. C. Savelkoul P. H. (2003). Use of amplified fragment length polymorphism analysis to identify medically important *Candida* spp., including *C. dubliniensis*. J. Clin. Microbiol. 41, 1357–1362. 10.1128/JCM.41.4.1357-1362.2003 12682114 PMC153876

[B9] Bougnoux M. E. Tavanti A. Bouchier C. Gow N. A. Magnier A. Davidson A. D.. (2003). Collaborative consensus for optimized multilocus sequence typing of *Candida albicans*. J. Clin. Microbiol. 41, 5265–5266. 10.1128/JCM.41.11.5265-5266.2003 14605179 PMC262540

[B10] Chowdhary A. Anil Kumar V. Sharma C. Prakash A. Agarwal K. Babu R.. (2014). Multidrug-resistant endemic clonal strain of *Candida auris* in India. Eur. J. Clin. Microbiol. Infect. Dis. 33, 919–926. 10.1007/s10096-013-2027-1 24357342

[B11] Cliff P. R. Sandoe J. A. Heritage J. Barton R. C. (2008). Use of multilocus sequence typing for the investigation of colonisation by *Candida albicans* in intensive care unit patients. J. Hosp. Infect. 69, 24–32. 10.1016/j.jhin.2008.02.006 18396349

[B12] Clinical Laboratory Standards Institute (2012). Reference Method for Broth Dilution Antifungal Susceptibility Testing of Yeasts; Fourth Informational Supplement, M27-S4. Wayne, PA: Clinical and Laboratory Standards Institute.

[B13] Escribano P. Rodríguez-Créixems M. Sánchez-Carrillo C. Muñoz P. Bouza E. Guinea J. (2013). Endemic genotypes of *Candida albicans* causing fungemia are frequent in the hospital. J. Clin. Microbiol. 51, 2118–2123. 10.1128/JCM.00516-13 23616451 PMC3697706

[B14] Fitzpatrick M. A. Ozer E. A. Hauser A. R. (2016). Utility of whole-genome sequencing in characterizing *Acinetobacter* epidemiology and analyzing hospital outbreaks. J. Clin. Microbiol. 54, 593–612. 10.1128/JCM.01818-15 26699703 PMC4767972

[B15] Garcia-Hermoso D. Cabaret O. Lecellier G. Desnos-Ollivier M. Hoinard D. Raoux D.. (2007). Comparison of microsatellite length polymorphism and multilocus sequence typing for DNA-Based typing of *Candida albicans*. J. Clin. Microbiol. 45, 3958–3963. 10.1128/JCM.01261-07 17928418 PMC2168554

[B16] Ge S. H. Xie J. Xu J. Li J. Li D. M. Zong L. L.. (2012). Prevalence of specific and phylogenetically closely related genotypes in the population of *Candida albicans* associated with genital candidiasis in China. Fungal Genet. Biol. 49, 86–93. 10.1016/j.fgb.2011.10.006 22079546

[B17] Hoben A. D. Buunk A. P. Fincher C. L. Thornhill R. Schaller M. (2010). On the adaptive origins and maladaptive consequences of human inbreeding: parasite prevalence, immune functioning, and consanguineous marriage. Evol. Psychol. 8, 658–676. 10.1177/147470491000800408 22947825 PMC10480803

[B18] Huyke J. Martin R. Walther G. Weber M. Kaerger K. Bougnoux M. E.. (2015). *Candida albicans* bloodstream isolates in a German university hospital are genetically heterogenous and susceptible to commonly used antifungals. Int. J. Med. Microbiol. 305, 742–747. 10.1016/j.ijmm.2015.08.027 26324013

[B19] Illnait-Zaragozí M. T. Martínez-Machín G. F. Fernández-Andreu C. M. Perurena-Lancha M. R. Theelen B. Boekhout T.. (2012). Environmental isolation and characterisation of *Cryptococcus* species from living trees in Havana city, Cuba. Mycoses 55, e138–e144. 10.1111/j.1439-0507.2012.02168.x 22364253

[B20] Kett D. H. Azoulay E. Echeverria P. M. Vincent J. L. (2011). Extended Prevalence of Infection in ICU Study (EPIC II) Group of Investigators. *Candida bloodstream* infections in intensive care units: analysis of the extended prevalence of infection in intensive care unit study. Crit. Care Med. 39, 665–670. 10.1097/CCM.0b013e318206c1ca 21169817

[B21] Kumamoto C. A. (2011). Inflammation and gastrointestinal *Candida* colonization. Curr. Opin. Microbiol. 14, 386–391. 10.1016/j.mib.2011.07.015 21802979 PMC3163673

[B22] Kwong J. C. Mercoulia K. Tomita T. Easton M. Li H. Y. Bulach D. M.. (2016). Prospective whole-genome sequencing enhances national surveillance of Listeria monocytogenes. J. Clin. Microbiol. 54, 333–342. 10.1128/JCM.02344-15 26607978 PMC4733179

[B23] Leroy O. Gangneux J. P. Montravers P. Mira J. P. Gouin F. Sollet J. P.. (2009). Epidemiology, management, and risk factors for death of invasive *Candida* infections in critical care: a multicenter, prospective, observational study in France (2005–2006). Crit. Care Med. 37, 1612–1618. 10.1097/CCM.0b013e31819efac0 19325476

[B24] Liu M. Huang S. Guo L. Li H. Wang F. Zhang Q. I.. (2015). Clinical features and risk factors for blood stream infections of *Candida* in neonates. Exp. Ther. Med. 10, 1139–1144. 10.3892/etm.2015.2626 26622453 PMC4533177

[B25] Long S. W. Beres S. B. Olsen R. J. Musser J. M. (2014). Absence of patient-to-patient intrahospital transmission of *Staphylococcus aureus* as determined by whole-genome sequencing. MBio 5, e01692–14. 10.1128/mBio.01692-14 25293757 PMC4196229

[B26] Maganti H. Yamamura D. Xu J. (2011). Prevalent nosocomial clusters among causative agents for candidemia in Hamilton, Canada. Med. Mycol. 49, 530–538. 10.3109/13693786.2010.547880 21198348

[B27] Marcos-Zambrano L. J. Escribano P. Sanguinetti M. Gómez G de la Pedrosa E. De Carolis E. Vella A.. (2015). Clusters of patients with candidaemia due to genotypes of *Candida albicans* and *Candida parapsilosis*: differences in frequency between hospitals. Clin. Microbiol. Infect. 21, 677–683. 10.1016/j.cmi.2015.03.007 25882359

[B28] McBryde E. S. Bradley L. C. Whitby M. McElwain D. L. (2004). An investigation of contact transmission of methicillin-resistant *Staphylococcus aureus*. J. Hosp. Infect. 58, 104–108. 10.1016/j.jhin.2004.06.010 15474180

[B29] McManus B. A. Coleman D. C. (2014). Molecular epidemiology, phylogeny and evolution of *Candida albicans*. Infect. Genet. Evol. 21, 166–178. 10.1016/j.meegid.2013.11.008 24269341

[B30] Moorhouse A. J. Rennison C. Raza M. Lilic D. Gow N. A. (2016). Clonal strain persistence of *Candida albicans* isolates from chronic mucocutaneous candidiasis patients. PLoS ONE 11:e0145888. 10.1371/journal.pone.0145888 26849050 PMC4743940

[B31] Moran G. P. Coleman D. C. Sullivan D. J. (2012). *Candida albicans* versus *Candida dubliniensis*: why is *C. albicans* more pathogenic? Int. J. Microbiol. 2012:205921. 10.1155/2012/205921 21904553 PMC3166774

[B32] Neofytos D. Fishman J. A. Horn D. Anaissie E. Chang C. H. Olyaei A.. (2010). Epidemiology and outcome of invasive fungal infections in solid organ transplant recipients. Transpl. Infect. Dis. 12, 220–229. 10.1111/j.1399-3062.2010.00492.x 20113459

[B33] Nübel U. Nachtnebel M. Falkenhorst G. Benzler J. Hecht J. Kube M.. (2013). MRSA transmission on a neonatal intensive care unit: epidemiological and genome-based phylogenetic analyses. PLoS ONE 8:e54898. 10.1371/journal.pone.0054898 23382995 PMC3561456

[B34] Odds F. C. Bougnoux M. E. Shaw D. J. Bain J. M. Davidson A. D. Diogo D.. (2007). Molecular phylogenetics of *Candida albicans*. Eukaryot. Cell 6, 1041–1052. 10.1128/EC.00041-07 17416899 PMC1951527

[B35] Odds F. C. Jacobsen M. D. (2008). Multilocus sequence typing of pathogenic *Candida* species. Eukaryot. Cell 7, 1075–1084. 10.1128/EC.00062-08 18456859 PMC2446668

[B36] Pfaller M. A. Moet G. J. Messer S. A. Jones R. N. Castanheira M. (2011). Geographic variations in species distribution and echinocandin and azole antifungal resistance rates among *Candida* bloodstream infection isolates: report from the SENTRY Antimicrobial Surveillance Program (2008 to 2009). J. Clin. Microbiol. 49, 396–399. 10.1128/JCM.01398-10 21068282 PMC3020436

[B37] Price J. R. Golubchik T. Cole K. Wilson D. J. Crook D. W. Thwaites G. E.. (2014). Whole-genome sequencing shows that patient-to-patient transmission rarely accounts for acquisition of *Staphylococcus aureus* in an intensive care unit. Clin. Infect. Dis. 58, 609–618. 10.1093/cid/cit807 24336829 PMC3922217

[B38] Roisin S. Gaudin C. De Mendonça R. Bellon J. Van Vaerenbergh K. De Bruyne K.. (2016). Pan-genome multilocus sequence typing and outbreak-specific reference-based single nucleotide polymorphism analysis to resolve two concurrent *Staphylococcus aureus* outbreaks in neonatal services. Clin. Microbiol. Infect. 22, 520–526. 10.1016/j.cmi.2016.01.024 26899827

[B39] Sampaio P. Gusmão L. Correia A. Alves C. Rodrigues A. G. Pina-Vaz C.. (2005). New microsatellite multiplex PCR for *Candida albicans* strain typing reveals microevolutionary changes. J. Clin. Microbiol. 43, 3869–3876. 10.1128/JCM.43.8.3869-3876.2005 16081924 PMC1233915

[B40] Schelenz S. Hagen F. Rhodes J. L. Abdolrasouli A. Chowdhary A. Hall A.. (2016). First hospital outbreak of the globally emerging *Candida auris* in a European hospital. Antimicrob. Resist. Infect. Control. 5:35. 10.1186/s13756-016-0132-5 27777756 PMC5069812

[B41] SenGupta D. J. Cummings L. A. Hoogestraat D. R. Butler-Wu S. M. Shendure J. Cookson B. T.. (2014). Whole-genome sequencing for high-resolution investigation of methicillin-resistant *Staphylococcus aureus* epidemiology and genome plasticity. J. Clin. Microbiol. 52, 2787–2796. 10.1128/JCM.00759-14 24850346 PMC4136142

[B42] Shin J. H. Bougnoux M. E. d'Enfert C. Kim S. H. Moon C. J. Joo M. Y.. (2011). Genetic diversity among Korean *Candida albicans* bloodstream isolates: assessment by multilocus sequence typing and restriction endonuclease analysis of genomic DNA by use of BssHII. J. Clin. Microbiol. 49, 2572–2577. 10.1128/JCM.02153-10 21562112 PMC3147862

[B43] Song E. S. Shin J. H. Jang H. C. Choi M. J. Kim S. H. Bougnoux M. E.. (2014). Multilocus sequence typing for the analysis of clonality among *Candida albicans* strains from a neonatal intensive care unit. Med. Mycol. 52, 653–658. 10.1093/mmy/myu028 25037934

[B44] Stokes C. Moran G. P. Spiering M. J. Cole G. T. Coleman D. C. Sullivan D. (2007). Lower filamentation rates of *Candida dubliniensis* contribute to its lower virulence in comparison with *Candida albicans*. Fungal Genet. Biol. 44, 920–931. 10.1016/j.fgb.2006.11.014 17251042

[B45] Sun H. Chen Y. Zou X. Li H. Yin X. Qin H.. (2016). Occurrence of oral *Candida* colonization and its risk factors among patients with malignancies in China. Clin. Oral Investig. 20, 459–467. 10.1007/s00784-015-1524-2 26220512

[B46] Tavanti A. Davidson A. D. Fordyce M. J. Gow N. A. Maiden M. C. Odds F. C. (2005). Population structure and properties of *Candida albicans*, as determined by multilocus sequence typing. J. Clin. Microbiol. 43, 5601–5613. 10.1128/JCM.43.11.5601-5613.2005 16272493 PMC1287804

[B47] Tavanti A. Gow N. A. Senesi S. Maiden M. C. Odds F. C. (2003). Optimization and validation of multilocus sequence typing for *Candida albicans*. J. Clin. Microbiol. 41, 3765–3776. 10.1128/JCM.41.8.3765-3776.2003 12904388 PMC179823

[B48] Tsai M. H. Wang S. H. Hsu J. F. Lin L. C. Chu S. M. Huang H. R.. (2015). Clinical and molecular characteristics of bloodstream infections caused by *Candida albicans* in children from 2003 to 2011. Clin. Microbiol. Infect. 21, e1–e8. 10.1016/j.cmi.2015.06.024 26148466

[B49] Ugolotti E. Larghero P. Vanni I. Bandettini R. Tripodi G. Melioli G.. (2016). Whole-genome sequencing as standard practice for the analysis of clonality in outbreaks of meticillin-resistant *Staphylococcus aureus* in a paediatric setting. J. Hosp. Infect. 93, 375–381. 10.1016/j.jhin.2016.04.003 27184087

[B50] Vincent J. L. Rello J. Marshall J. Silva E. Anzueto A. Martin C. D.. (2009). International study of the prevalence and outcomes of infection in intensive care units. JAMA 302, 2323–2329. 10.1001/jama.2009.1754 19952319

[B51] Wu K. Luo T. Li L. Zhang Q. Zhu J. Gao Q.. (2015). Multilocus sequence typing of pathogenic *Candida albicans* isolates collected from a teaching hospital in Shanghai, China: a molecular epidemiology study. PLoS ONE 28:e0125245. 10.1371/journal.pone.0125245 25919124 PMC4412568

[B52] Zomorodian K. Haghighi N. N. Rajaee N. Pakshir K. Tarazooie B. Vojdani M.. (2011). Assessment of *Candida* species colonization and denture-related stomatitis in complete denture wearers. Med. Mycol. 49, 208–211. 10.3109/13693786.2010.507605 20795762

