# Supplementary material for: Molecular Fingerprinting Studies Do Not Support Intrahospital Transmission of Candida albicans among Candidemia Patients in Kuwait
Source: Front Microbiol. 2017 Feb 21;8:247. doi: 10.3389/fmicb.2017.00247 (PMC5318450; doi:10.3389/fmicb.2017.00247)
Supplement: Supplementary file 1 [file Table1.DOCX]

**Supplementary Table 1. Demographic and other details of candidemia patients and fingerprinting data for 102 *C. albicans* isolates from Kuwait by MLST**

| **Patient's demographic data** | | | **Name of** | **Admission** | **Isolate** | **Date of isolation** | **Fingerprinting by MLST** | | |
| --- | --- | --- | --- | --- | --- | --- | --- | --- | --- |
| **Serial no.** | **Age** | **Nationality** | **Hospital** | **to ICU** | **no.** | **(DD-MM-YY)** | **DST** | **Isolate in cluster** | **Cluster no.** |
| 1 | NB | Kuwaiti | Maternity | Yes | Kw31 | 01-01-11 | 2316 | No | NA |
| 2 | 47 Y | Kuwaiti | Mubarak Al-Kabeer | No | Kw146 | 08-01-11 | 461 | No | NA |
| 3 | 49 Y | Indian | Mubarak Al-Kabeer | No | Kw177 | 13-01-11 | 1564 | No | NA |
| 4 | NB | Kuwaiti | Maternity | Yes | Kw458 | 30-01-11 | 918 | No | NA |
| 5 | 66 Y | Kuwaiti | Amiri | Yes | Kw1104 | 25-03-11 | 1056 | No | NA |
| 6 | 61 Y | Kuwaiti | Al-Sabah | Yes | Kw891 | 26-03-11 | 656 | Yes | I |
| 7 | 22 D | Kuwaiti | Maternity | Yes | Kw1158 | 27-03-11 | 601 | Yes | VI |
| 8 | 1 M | Kuwaiti | Farwaniya | Yes | Kw1275 | 05-04-11 | 620 | Yes | IX |
| 9 | 69 Y | Kuwaiti | Mubarak Al-Kabeer | No | Kw1311 | 09-04-11 | 840 | Yes | V |
| 10 | 78 Y | Kuwaiti | Mubarak Al-Kabeer | Yes | Kw1342 | 11-04-11 | 1133 | Yes | XII |
| 11 | 79 Y | Kuwaiti | Amiri | Yes | Kw1443 | 18-04-11 | 840 | Yes | V |
| 12 | NB | Kuwaiti | Maternity | Yes | Kw1703 | 12-05-11 | 565 | No | NA |
| 13 | NB | Indian | Ibn-Sina | Yes | Kw1660 | 12-05-11 | 2285 | No | NA |
| 14 | 68 Y | Kuwaiti | Mubarak Al-Kabeer | No | Kw1894 | 30-05-11 | 2286 | Yes | XIV |
| 15 | 2 D | Kuwaiti | Al-Sabah | Yes | Kw2019 | 14-06-11 | 656 | Yes | I |
| 16 | 61 Y | Kuwaiti | Mubarak Al-Kabeer | No | Kw2079 | 17-06-11 | 656 | Yes | I |
| 17 | 63 Y | Kuwaiti | Farwaniya | Yes | Kw2247 | 04-07-11 | 659 | Yes | IV |
| 18 | 65 Y | Kuwaiti | Mubarak Al-Kabeer | No | Kw2256 | 04-07-11 | 1717 | Yes | III |
| 19 | 10 D | Kuwaiti | Farwaniya | Yes | Kw2283 | 04-07-11 | 2286 | Yes | XIV |
| 20 | NB | Kuwaiti | Jahra | No | Kw2227 | 04-07-11 | 2287 | No | NA |
| 21 | NB | Kuwaiti | Jahra | Yes | Kw2306 | 06-07-11 | 2288 | No | NA |
| 22 | 38 Y | Kuwaiti | Mubarak Al-Kabeer | No | Kw2444 | 23-07-11 | 777 | No | NA |
| 23 | 3 M | Kuwaiti | Farwaniya | Yes | Kw2418 | 23-07-11 | 2289 | Yes | XV |
| 24 | 30 Y | Kuwaiti | Maternity | Yes | Kw2558 | 30-07-11 | 1097 | Yes | XI |
| 25 | 1 M | Kuwaiti | Ibn-Sina | No | Kw2545 | 09-08-11 | 2290 | No | NA |
| 26 | 71 Y | Egyptian | Ibn-Sina | Yes | Kw2618 | 10-08-11 | 2291 | No | NA |
| 27 | 29 Y | Kuwaiti | Mubarak Al-Kabeer | No | Kw2603 | 10-08-11 | 1717 | Yes | III |
| 28 | 15 D | Kuwaiti | Maternity | Yes | Kw2728 | 21-08-11 | 2292 | Yes | XVI |
| 29 | 33 D | Kuwaiti | Mubarak Al-Kabeer | Yes | Kw2691 | 24-08-11 | 2289 | Yes | XV |
| 30 | 46 Y | Kuwaiti | Ibn-Sina | Yes | Kw2765 | 06-09-11 | 656 | Yes | I |
| 31 | 8 Y | Kuwaiti | Mubarak Al-Kabeer | No | Kw2784 | 06-09-11 | 656 | Yes | I |
| 32 | NB | Kuwaiti | Maternity | Yes | Kw3210 | 09-09-11 | 2294 | No | NA |
| 33 | 13 D | Kuwaiti | Maternity | Yes | Kw3039 | 18-09-11 | 601 | Yes | VI |
| 34 | 65 Y | Kuwaiti | Ibn-Sina | Yes | Kw2971 | 22-09-11 | 124 | No | NA |
| 35 | 2 M | Kuwaiti | Al-Sabah | Yes | Kw3003 | 28-09-11 | 2293 | No | NA |
| 36 | 53 Y | Kuwaiti | Al-Sabah | No | Kw3136 | 03-10-11 | 3054 | No | NA |
| 37 | 78 Y | Kuwaiti | Amiri | Yes | Kw3037 | 04-10-11 | 1631 | No | NA |
| 38 | 71 Y | Kuwaiti | Mubarak Al-Kabeer | No | Kw3541 | 13-10-11 | 620 | Yes | IX |
| 39 | 10 D | Indian | Maternity | Yes | Kw3306 | 13-10-11 | 601 | Yes | VI |
| 40 | NB | Kuwaiti | Maternity | Yes | Kw3491 | 14-10-11 | 605 | No | NA |
| 41 | 63 Y | Kuwaiti | Amiri | No | Kw3690 | 15-10-11 | 2297 | No | NA |
| 42 | 43 Y | Kuwaiti | Ibn-Sina | No | Kw3196 | 16-10-11 | 656 | Yes | I |
| 43 | 76 Y | Kuwaiti | Al-Sabah | No | Kw3276 | 21-10-11 | 1133 | Yes | XII |
| 44 | 106 Y | Kuwaiti | Jahra | No | Kw3376 | 27-10-11 | 2292 | Yes | XVI |
| 45 | 64 Y | Kuwaiti | Ibn-Sina | Yes | Kw3358 | 27-10-11 | 2295 | No | NA |
| 46 | 10 M | Kuwaiti | Jahra | No | Kw3410 | 28-10-11 | 2315 | No | NA |
| 47 | 60 Y | Kuwaiti | Farwaniya | No | Kw3427 | 29-10-11 | 621 | No | NA |
| 48 | NB | Egyptian | Maternity | Yes | Kw3442 | 01-11-11 | 79 | Yes | II |
| 49 | NB | Kuwaiti | Maternity | Yes | Kw3489 | 01-11-11 | 79 | Yes | II |
| 50 | 1 Y | Kuwaiti | Farwaniya | Yes | Kw3484 | 02-11-11 | 2296 | No | NA |
| 51 | 35 Y | Kuwaiti | Jahra | No | Kw3766 | 03-12-11 | 1097 | Yes | XI |
| 52 | 69 Y | Kuwaiti | Amiri | Yes | Kw3772 | 04-12-11 | 1110 | No | NA |
| 53 | 16 D | Kuwaiti | Maternity | Yes | Kw34 | 20-12-11 | 1865 | No | NA |
| 54 | 4 D | Kuwaiti | Ibn-Sina | No | Kw195 | 16-01-12 | 656 | Yes | I |
| 55 | 42 Y | Kuwaiti | Ibn-Sina | No | Kw255 | 19-01-12 | 2116 | No | NA |
| 56 | 15 D | Kuwaiti | Maternity | Yes | Kw277-12 | 19-01-12 | 747 | No | NA |
| 57 | 41 Y | Kuwaiti | Amiri | No | Kw431 | 30-01-12 | 90 | No | NA |
| 58 | 1 M | Kuwaiti | Farwaniya | Yes | Kw541 | 09-02-12 | 840 | Yes | V |
| 59 | 38 Y | Kuwaiti | Al-Sabah | No | Kw788 | 09-02-12 | 1613 | No | NA |
| 60 | 49 Y | Kuwaiti | Al-Sabah | No | Kw554 | 10-02-12 | 656 | Yes | I |
| 61 | 62 Y | Kuwaiti | Mubarak Al-Kabeer | Yes | Kw909 | 12-02-12 | 2319 | No | NA |
| 62 | 42 D | Kuwaiti | Maternity | Yes | Kw606 | 13-02-12 | 277 | Yes | VII |
| 63 | 35 Y | Egyptian | Mubarak Al-Kabeer | No | Kw575 | 13-02-12 | 1363 | Yes | XIII |
| 64 | 50 Y | Kuwaiti | Ibn-Sina | No | Kw811 | 02-03-12 | 659 | Yes | IV |
| 65 | 54 Y | Kuwaiti | Mubarak Al-Kabeer | No | Kw68-12 | 06-04-12 | 2307 | No | NA |
| 66 | 46 Y | Kuwaiti | Mubarak Al-Kabeer | No | Kw70 | 06-04-12 | 2309 | No | NA |
| 67 | 53 Y | Kuwaiti | Jahra | No | Kw247 | 15-04-12 | 2317 | No | NA |
| 68 | 48 Y | Iranian | Farwaniya | No | Kw316 | 26-04-12 | 725 | No | NA |
| 69 | 56 Y | Kuwaiti | Mubarak Al-Kabeer | No | Kw203 | 18-05-12 | 2300 | No | NA |
| 70 | 42 Y | Kuwaiti | Ibn-Sina | No | Kw262 | 25-05-12 | 927 | Yes | X |
| 71 | 49 Y | Kuwaiti | Mubarak Al-Kabeer | No | Kw286 | 28-05-12 | 1763 | No | NA |
| 72 | 15 D | Kuwaiti | Jahra | Yes | Kw77 | 08-06-12 | 277 | Yes | VII |
| 73 | 3 D | Kuwaiti | Maternity | Yes | Kw278 | 28-06-12 | 79 | Yes | II |
| 74 | 55 Y | Kuwaiti | Farwaniya | No | Kw20 | 03-07-12 | 2299 | No | NA |
| 75 | 48 Y | Kuwaiti | Mubarak Al-Kabeer | Yes | Kw98 | 09-07-12 | 1363 | Yes | XIII |
| 76 | 10 D | Kuwaiti | Maternity | Yes | Kw287 | 23-07-12 | 927 | Yes | X |
| 77 | 39 Y | Egyptian | Farwaniya | No | Kw332 | 25-07-12 | 3053 | No | NA |
| 78 | 42 Y | Kuwaiti | Mubarak Al-Kabeer | No | Kw340 | 26-07-12 | 3055 | No | NA |
| 79 | 68 Y | Kuwaiti | Mubarak Al-Kabeer | Yes | Kw329 | 30-07-12 | 2302 | No | NA |
| 80 | 49 Y | Indian | Ibn-Sina | No | Kw341 | 30-09-12 | 2304 | No | NA |
| 81 | 61 Y | Kuwaiti | Ibn-Sina | No | Kw94 | 05-10-12 | 3052 | No | NA |
| 82 | 63 Y | Kuwaiti | Mubarak Al-Kabeer | No | Kw75 | 08-10-12 | 2310 | No | NA |
| 83 | 35 D | Indian | Maternity | Yes | Kw284 | 13-10-12 | 1717 | Yes | III |
| 84 | 47 Y | Kuwaiti | Amiri | No | Kw180 | 19-11-12 | 2298 | No | NA |
| 85 | 19 D | Kuwaiti | Maternity | Yes | Kw156 | 23-11-12 | 364 | No | NA |
| 86 | 40 Y | Kuwaiti | Mubarak Al-Kabeer | No | Kw107 | 07-12-12 | 79 | Yes | II |
| 87 | 44 Y | Kuwaiti | Ibn-Sina | No | Kw73 | 07-12-12 | 1717 | Yes | III |
| 88 | 38 Y | Kuwaiti | Mubarak Al-Kabeer | No | Kw216 | 16-12-12 | 365 | Yes | VIII |
| 89 | 38 Y | Kuwaiti | Jahra | No | Kw240 | 21-12-12 | 37 | No | NA |
| 90 | 57 Y | Kuwaiti | Ibn-Sina | No | Kw367 | 31-12-12 | 2305 | No | NA |
| 91 | 46 Y | Filipino | Ibn-Sina | No | Kw366 | 27-01-13 | 659 | Yes | IV |
| 92 | 34 Y | Kuwaiti | Al-Sabah | No | Kw106 | 06-02-13 | 590 | No | NA |
| 93 | 46 Y | Kuwaiti | Mubarak Al-Kabeer | No | Kw87 | 05-03-13 | 2323 | No | NA |
| 94 | 21 D | Kuwaiti | Maternity | Yes | Kw415 | 27-03-13 | 2140 | No | NA |
| 95 | 39 Y | Kuwaiti | Mubarak Al-Kabeer | No | Kw288 | 18-04-13 | 656 | Yes | I |
| 96 | 34 Y | Kuwaiti | Farwaniya | No | Kw67 | 04-05-13 | 1299 | No | NA |
| 97 | 42 Y | Kuwaiti | Farwaniya | No | Kw68-13 | 05-05-13 | 2314 | No | NA |
| 98 | NB | Kuwaiti | Maternity | Yes | Kw91 | 08-05-13 | 2322 | No | NA |
| 99 | 60 | Kuwaiti | Mubarak Al-Kabeer | No | Kw109 | 06-07-13 | 913 | No | NA |
| 100 | 15 D | Kuwaiti | Farwaniya | No | Kw276 | 18-07-13 | 365 | Yes | VIII |
| 101 | 55 Y | Kuwaiti | Ibn-Sina | Yes | Kw2736 | 20-07-13 | 659 | Yes | IV |
| 102 | 45 Y | Egyptian | Farwaniya | No | Kw277-13 | 18-07-13 | 2313 | No | NA |

^a^NB, new born; D, days, M, months; Y, years

^b^Cluster isolates are numbered in Roman numerals. NA, not applicable

New DSTs detected in this study are underlined
