# Supplementary figures and images for: Molecular Fingerprinting Studies Do Not Support Intrahospital Transmission of Candida albicans among Candidemia Patients in Kuwait
Source: Front Microbiol. 2017 Feb 21;8:247. doi: 10.3389/fmicb.2017.00247 (PMC5318450; doi:10.3389/fmicb.2017.00247)

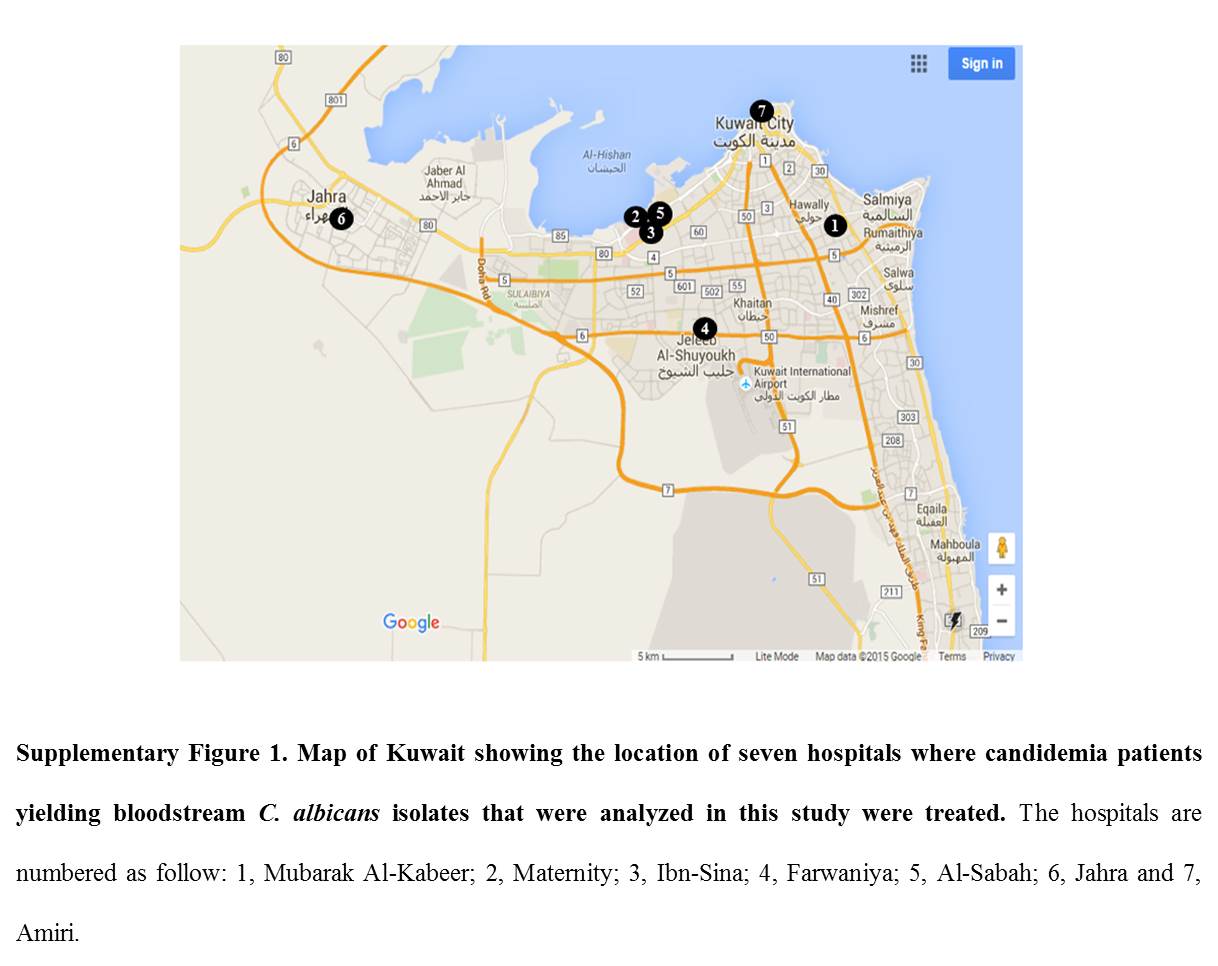

Supplement: Supplementary file 2 [file Image1.JPEG]
